# Role of Esketamine in Attenuating Neuroinflammation and Improving Postoperative Cognitive Function via Autophagy Activation Through PARP1 Targeting

**DOI:** 10.33549/physiolres.935577

**Published:** 2025-10-01

**Authors:** Chendi ZHAO, Jiaxin LIU, Shuang ZHAO, Peng LIU, Zhao LI, Yongle LI, Rui DONG, Xiu-Li WANG

**Affiliations:** 1 Department of Anesthesiology, The Third Hospital of Hebei Medical University, Shijiazhuang, China; 2 Department of Anesthesiology, Affiliated Hospital of Hebei University, Baoding, China; 3 Department of Anesthesiology, The First Hospital of Qinhuangdao, Qinhuangdao, China

**Keywords:** Autophagy, Esketamine, Neuroinflammation, PARP1, Postoperative cognitive dysfunction

## Abstract

Postoperative cognitive dysfunction (POCD) substantially influences patient outcomes, with its pathophysiology potentially linked to neuroinflammation induced by surgical procedures and anesthesia. Previous research has indicated that esketamine may alleviate neuroinflammation. Therefore, elucidating the mechanisms through which esketamine modulates neuroinflammation to ameliorating POCD is crucial for advancing its clinical management. An *in vivo* model of POCD was established using C57BL/6J mice subjected to exploratory laparotomy. Cognitive performance was evaluated through the Morris water maze. Subsequently, hippocampal tissue samples were collected to measure changes in the levels of IL-1β, IL-6, TNF-α, PARP1, SIRT1, LC3, and P62. *In vitro* experiments were performed using BV2 microglial cells treated with lipopolysaccharides (LPS) to induce inflammation and a PARP1 plasmid to create PARP1 overexpression (OvPARP1) models. These models were treated with esketamine, followed by assessment of changes in the previously mentioned indicators. Immunofluorescence microscopy was used to examine PARP1 expression, while transmission electron microscopy was used to analyze cellular autophagy. Exploratory laparotomy induced POCD and triggered neuroinflammation within the hippocampus of the mice. Treatment with esketamine alleviated POCD by inhibiting OvPARP1 expression and increasing SIRT1 levels, which promoted cellular autophagy and reduced neuroinflammation. Esketamine regulates the PARP1-SIRT1 pathway, thereby activating autophagy, reducing neuroinflammation, and improving POCD. These findings provide novel insights into potential therapeutic strategies for the management of POCD.

## Introduction

Postoperative cognitive dysfunction (POCD) is a frequently encountered neurological complication during the perioperative period. It is characterized by reduced memory, slowed movement, extended hospitalization, and increased medical costs for patients [1]. As a result, POCD has emerged as a key area of research in recent years. Neuroinflammation is closely linked to cognitive impairment, and clinical trials have demonstrated that reducing neuroinflammation can decrease the incidence of POCD [2–4]. Despite extensive research on neuroinflammation, the endogenous mechanisms by which it is regulated to enhance cognitive outcomes remain inadequately understood.

Esketamine, the dextrorotatory isomer of ketamine, has gained the attention of researchers, with earlier studies primarily focusing on its roles in anxiety treatment and analgesia [5]. More recently, there has been growing interest in the impact of esketamine on cognitive function. Studies have indicated that esketamine can enhance early postoperative cognitive function in older adult patients [6]. Additionally, it has been observed that esketamine can partially reduce the production of pro-inflammatory cytokines [7]. Moreover, esketamine ameliorates POCD through modulation of the BDNF-TrkB signaling pathway, and can mitigate LPS-induced neuroinflammation by inhibiting BDNF signal transduction [8,9]. Furthermore, esketamine suppresses microglial activation and decreases the expression of inflammatory mediators [10]. In summary, current evidence indicates that the neuroprotective effects of esketamine are primarily associated with its capacity to reduce microglial activation and alleviate neuroinflammation.

Microglia, as resident immune cells within the central nervous system, serve as key effectors in mediating neurogenic inflammatory responses in the brain. They play a key role in the regulation of neuroinflammation and cognitive neural functions [11]. Surgical trauma can lead to the overactivation of microglia, resulting in the release of pro-inflammatory cytokines, which is a major contributor to the onset and progression of POCD [12,13]. Research reveals the involvement of PARP1 in the inflammatory processes of microglia [14]. However, it remains unclear whether esketamine can mitigate neuroinflammation through the modulation of PARP1.

Autophagy, also known as type II programmed cell death, is crucial in various pathophysiological processes, including cellular homeostasis, aging, immune responses, tumorigenesis, and neurodegenerative diseases. Studies have indicated that promoting autophagy in microglia reduces neuroinflammation [15]. PARP1 inhibits SIRT1-mediated autophagy by depleting NAD^+^ levels [16–18]. The potential of modulating PARP1 levels in microglia to influence autophagy and subsequently alleviate neuroinflammation remains to be fully examined.

The objective of this study is to examine the role of PARP1 in regulating neuroinflammation, using *in vivo* and *in vitro* experiments to demonstrate that PARP1 could serve as a therapeutic target for esketamine in reducing neuroinflammation. The findings reveal that esketamine can enhance autophagy, thereby alleviating neuroinflammation and improving POCD in mice through the regulation of PARP1 expression. These results indicate that PARP1 may represent a promising therapeutic target, providing new insights into the management of POCD and the development of strategies for postoperative neuroprotection.

## Materials and Methods

### Reagents and antibodies

LPS was procured from Sigma-Aldrich (L2630), and esketamine was supplied by Jiangsu Hengrui Pharmaceuticals Co., Ltd. (230623BL). Antibodies for PARP1 (Proteintech Group, Inc, 13371-1-AP), SIRT1 (Proteintech Group, Inc, 13161-1-AP), LC3 (Proteintech Group, Inc, 14600-1-AP), P62 (Proteintech Group, Inc, 18420-1-AP), GAPDH (Proteintech Group, Inc, 10494-1-AP), IL-6, and TNF-α (Proteintech Group, Inc, 26404-1-AP, 17590-1-AP) were obtained from Proteintech Group, Inc. IL-1β was acquired from Beijing Biosynthesis Biotechnology Co., Ltd. (bs-20449R). Secondary antibodies, including goat anti-rabbit IgG-FITC labeled, goat anti-mouse IgG-FITC labeled, and DAPI, were all sourced from Abcam (Ab7086, Ab6785, Ab285390).

The RFect plasmid DNA transfection kit was obtained from Changzhou Bio-Generating Biotechnology Corp (21014). The Coenzyme I (NAD^+^/NADH) content test kit was sourced from Nanjing Jiancheng Bioengineering Institute (A114-1).

### Plasmid

The OvPARP1 plasmid was obtained from Obio Technology (Shanghai, China).

### Animal model and grouping

Male C57BL/6J mice, aged 10 to 12 months, were obtained from SPF (Beijing) Biotechnology Co., Ltd. All experimental procedures received approval from the Experimental Animal Committee of Hebei University (Approval No.: IACUC-202326XR) and adhered to the guidelines for the care and use of laboratory animals. The mice were maintained in a controlled environment, with a temperature of 22±1 °C, relative humidity of 50±10 %, and a 12-hour light/dark cycle, with unrestricted access to food and water.

The mice were randomly assigned to four groups, each consisting of 6 mice: (1) Ctrl group, serving as the blank control group; (2) N group, non-surgery group, where mice were exposed to anesthesia only, without undergoing surgery; (3) S group, where mice received anesthesia followed by surgery; and (4) SE group, which involved esketamine pretreatment prior to anesthesia and subsequent surgery.

### Experimental methods and surgical model

Following a period of acclimatization, the mice underwent the water maze place navigation test. After this test was completed, drug pretreatment was administered. The SE group received an intraperitoneal injection of esketamine at a dose of 5 mg/kg (esketamine diluted with saline to 5 mg/l), while the other three groups were administered an equivalent volume of saline intraperitoneally. Thirty minutes after drug administration, the mice underwent model construction. Three days post-surgery, the water maze spatial probe test was conducted. One week after surgery, the mice were deeply anesthetized with pentobarbital sodium, decapitated quickly, and their hippocampal tissue was collected for further analysis.

The POCD model in aged mice was established through exploratory laparotomy under isoflurane anesthesia, following a method adapted from the work of Li *et al.* with slight modifications [19]. Mice were first placed in an anesthesia chamber containing 1.5 % isoflurane and 2 l/min oxygen, connected to an anesthesia machine (RWD Life Science Co., Ltd., Shenzhen, China) and a waste gas absorption system. After achieving anesthesia, the mice were positioned on a heating pad, with anesthesia maintained through a face mask inhalation of isoflurane. The entire surgical procedure lasted approximately 30 min. The abdominal hair was removed, and the area was disinfected with iodophor. A midline incision of approximately 1 cm was made in the abdomen, penetrating the peritoneal cavity. The liver, spleen, stomach, kidneys, and small intestine were sequentially examined using sterile cotton swabs. Finally, the peritoneum, muscle layer, and skin were sutured using absorbable sutures. The incision site was treated with 0.1 % lidocaine to reduce postoperative pain.

### Morris water maze test

The Morris water maze apparatus comprises of a circular water tank with a diameter of 120 cm and a depth of 60 cm, as well as a platform with a diameter of 6 cm. The entire area is divided into four quadrants. Prior to surgery, mice underwent a 5-day place navigation test. For this test, the platform was fixed in one quadrant, positioned at a height of 0.5 to 1 cm below the water surface. Mice were placed in the water, facing the pool wall, from each of the four quadrants, and the time taken to locate the platform was recorded. If a mouse failed to find the platform within 60 s, it was guided to the platform and allowed to remain there for 15 s [20]. Following the surgery, a spatial probe test was conducted. In this test, the platform was removed, and the mouse was placed in the water facing the pool wall from the quadrant opposite to the original platform location. Within a 60-second time frame, the escape latency (time taken to first reach the original platform location), the number of crossings over the original platform location, and the time spent in the quadrant where the platform was originally situated were recorded. All test results were documented using a video tracking system (SMART V3.0, RWD Life Science Co., Ltd.). In this experiment, the experimenters involved in evaluating the mouse behavior were completely unaware of the grouping treatment of the mice.

### Enzyme-linked immunosorbent assay

Enzyme-linked immunosorbent assay (ELISA) kits were used for the quantitative analysis of cytokine expression, specifically using IL-1β (MSKBIO, KT21178), IL-6 (MSKBIO, KT99854), and TNF-α (MSKBIO, KT99985) kits.

### Cell culture and group treatment

#### BV2 microglial cells (Procell, CL-0493)

Mouse BV2 microglial cells (Procell, CL-0493, Wuhan, China) were cultured in Dulbecco’s Modified Eagle Medium (DMEM) (Thermo Fisher Scientific (Gibco), Grand Island, New York) supplemented with 10 % FBS (Zhejiang Tianhang Biotechnology Co., Ltd., 11011-8611) and 1 % penicillin/streptomycin. Cells at 90 % confluence were collected, digested, and subsequently seeded into six-well plates at a density of 1×10^5^ cells/well. The study included the following experimental groups: (1) Ctrl group: cells were maintained in the original medium without additional treatment. (2) LPS group: culture medium was replaced with medium containing 1 μg/ml LPS for 24 h [21]. (3) LPS+Esk group: after 24 h of culture in medium containing 1 μg/ml LPS, the medium was replaced with medium containing 5 μg/ml esketamine for an additional 24 h. (4) OvPARP1 group: BV2 microglial cells were transfected with OvPARP1 plasmid and cultured for 24 h. (5) OvPARP1+Esk group: Following 24 h of OvPARP1 plasmid transfection, the medium was replaced with medium containing 5 μg/ml esketamine for another 24 h. (6) OvPARP1+RAPA group: After 24 h of OvPARP1 plasmid transfection, the medium was replaced with medium containing 100 nmol/l of the autophagy agonist RAPA for an additional 24 h.

#### Western blot

BV2 microglial cells or hippocampal tissue were lysed in cell lysis buffer (Cloud-Clone Corp., A102, Wuhan, China) on ice. The lysates were subsequently centrifuged at 12000 rpm for 10 min at 4 °C. The supernatant was collected, and the total protein content was determined using a Bicinchoninic Acid (BCA) assay kit (Cloud-Clone Corp., A202, Wuhan, China). Samples were then loaded into the wells of an SDS-PAGE gel, where they were separated and subsequently transferred to PVDF membranes. The membranes were blocked with skim milk overnight at 4 °C, followed by incubation with primary antibodies for 2 h at 37 °C. Afterward, the membranes were incubated with a secondary antibody working solution for 1 h at 37 °C. Results were visualized using an ECL chemiluminescence instrument and analyzed accordingly.

#### Immunofluorescence analysis

Cells were fixed with 4 % paraformaldehyde for 20 min and subsequently permeabilized with 0.25 % Triton X-100 (Sinopharm Group, 30188928) at room temperature for 30 min. Following permeabilization, cells were blocked with BSA (Sigma, A7030) at room temperature for 50 min. Cells were then incubated overnight at 4 °C with the anti-PARP1 antibody, followed by three washes with Phosphate-Buffered Saline (PBS). Subsequently, a secondary antibody was applied for 1 to 2 h at room temperature in the dark. Cell nuclei were stained with DAPI. All coverslips were mounted onto microscope slides using fluorescent mounting medium (Solarbio, S2100). All samples were analyzed using an inverted optical microscope (Olympus, Japan).

#### Transmission electron microscope observation

Cells were collected through centrifugation, resuspended in electron microscope fixative without culture medium, and fixed at 4 °C for 2 to 4 h. Following centrifugation, the supernatant was discarded, and 0.1 M phosphate buffer (PB) (pH 7.4) was added. The mixture was washed for 3 min and centrifuged again, repeating the washing process three times. The cell pellet was then suspended and encapsulated in a 1 % agarose solution. Samples were fixed with 1 % osmium tetroxide for 2 h at room temperature in the dark, washed three times with 0.1 M phosphate buffer (PB) (pH 7.4), and dehydrated using an ascending ethanol series (30 %-50 %-70 %-80 %-95 %-100 %-100 %), followed by two rounds of 100 % acetone. The samples were embedded in an epoxy resin embedding agent and polymerized at 60 °C for 48 h, after which they were cut into sections measuring 60 to 80 nm. Sections were stained in the dark with a 2 % uranyl acetate saturated alcohol solution for 8 min, followed by staining with a 2.6 % lead citrate solution for 8 min while avoiding CO_2_ exposure. After drying overnight at room temperature, samples were examined under a transmission electron microscope, and images were collected for analysis.

#### NAD^+^ detection

NAD^+^ levels in the Ctrl group, OvPARP1 group, and OvPARP1+Esk group were measured following the instructions provided by the manufacturer in the assay kit.

#### Statistical analysis

All experiments were conducted independently a minimum of three times. Data analysis and processing were performed using SPSS 21.0 (IBM) and GraphPad Prism 9.5.1 software. Measurement data are presented as mean ± standard deviation (SD) or mean ± standard error of the mean (SEM). Multiple comparisons were assessed using one-way ANOVA, followed by the Bonferroni *post hoc* test, while corrected one-way ANOVA used Dunnett’s method for pairwise comparisons. Changes in escape latency over time during the MWM place navigation phase were assessed using a separate two-way repeated measures ANOVA. P<0.05 was deemed statistically significant.

## Results

### Esketamine enhances postoperative cognitive function and reduces neuroinflammation in mice hippocampus

**Fig. 1:**
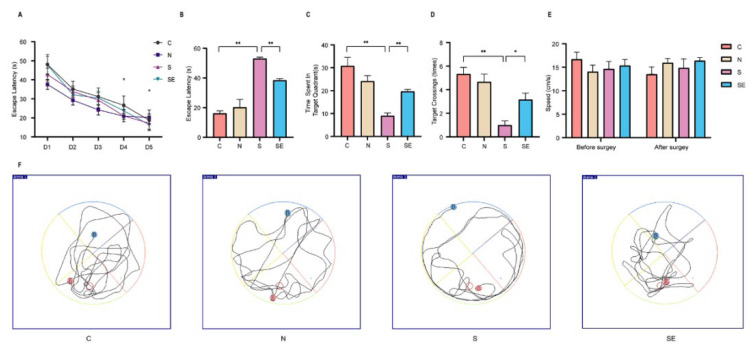
Esketamine alleviates cognitive dysfunction in mice after exploratory laparotomy. (**A**) Performance results of the place navigation test for each group of mice prior to exploratory laparotomy, assessing baseline spatial learning abilities. (**B**) Escape latency results during the spatial probe test for each group of mice following surgery, reflecting time taken to reach the target area. (**C**) Results of time spent in the target quadrant (the quadrant containing the original platform) during the spatial probe test for each group of mice post-surgery, indicating memory retention of the platform location. (**D**) Results of the number of crossings over the original platform location during the spatial probe test for each group of mice after surgery, representing memory recall accuracy. Data are expressed as mean ± standard error (SE), with * P<0.05 and ** P<0.01 indicating statistically significant differences. (**E**) Comparison of preoperative and postoperative swimming speed among different groups of mice. (**F**) Motion trajectory diagrams of the spatial probe test for each group of mice post-surgery, illustrating search patterns. (C group: control group, administered saline prior to surgery; N group: isoflurane anesthesia only without surgery, administered saline prior to surgery; S group: anesthesia and surgery, administered saline prior to surgery; SE group: anesthesia and surgery, administered esketamine prior to surgery).

To examine the effect of esketamine on postoperative cognitive function in mice, a water maze test was conducted. Male C57BL/6J mice were randomly assigned to four groups: control group (C group), non-surgery group (N group), surgery group (S group), and surgery with esketamine administration group (SE group), with six mice in each group. Based on prior research, exploratory laparotomy was used to construct a model of POCD. As depicted in Figure 1A, during the five consecutive days of the place navigation test preceding the exploratory laparotomy, the escape latency of mice in all groups progressively decreased as training continued, indicating an improvement in spatial learning ability with increased training duration. Figure 1B depicts that post-exploratory laparotomy, the escape latency for mice in the S group was significantly longer compared to the C group, while the SE group exhibited shorter escape latency than the S group (P<0.05). In the spatial probe test conducted after surgery, the S group demonstrated a significant reduction in the number of platform crossings compared to the C group, whereas the SE group had a greater number of crossings than the S group (P<0.05). The time spent in the target quadrant (E) mirrored the trends observed in the number of platform crossings, as depicted in Figure 1C and 1D. These results indicate that exploratory laparotomy results in a decline in spatial learning ability in mice, while pre-surgical administration of esketamine mitigate the adverse effects of surgery on learning ability. As shown in Figure 1E, there was no statistically significant difference in postoperative swimming speed among the groups of mice compared to preoperative levels. The decline in cognitive function in mice was not reflected in swimming speed. Figure 1F presents the swimming trajectories of mice during the postoperative water maze spatial exploration test, with the blue dot representing the starting point and the red dot representing the endpoint. Notably, mice in the S group exhibited chaotic swimming trajectories and longer paths to reach the target area compared to other groups. In contrast, mice in the SE group displayed shorter swimming trajectories to reach the target area when compared to the S group. Thus, these findings indicate that exploratory laparotomy reduces spatial memory in mice, while esketamine may enhance postoperative spatial memory.

Furthermore, studies have established a strong correlation between cognitive dysfunction and neuroinflammation [22]. To examine whether the cognitive improvements associated with esketamine are linked to reductions in neuroinflammation, ELISA was performed on the hippocampal tissue from the aforementioned experimental groups.

As shown in Table 1, no statistically significant differences were observed between the N and C groups, suggesting that isoflurane anesthesia does not provoke neuroinflammation. Conversely, the levels of inflammatory factors IL-1β, IL-6, and TNF-α in the S group were significantly elevated compared to the C group, while the SE group exhibited markedly lower expression levels of these inflammatory factors when compared to the S group. Taken together, these results indicate that exploratory laparotomy induces central nervous system inflammation in mice, and that esketamine may alleviate postoperative cognitive dysfunction by mitigating surgery-induced neuroinflammation.

### Esketamine can inhibit PARP1 expression and activate autophagy suppressed by exploratory laparotomy

**Fig. 2:**
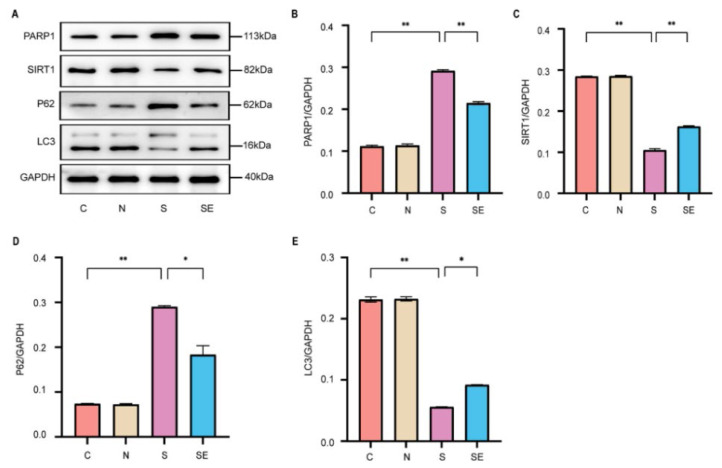
Esketamine inhibits PARP1 expression and activates autophagy in the mouse hippocampus. (**A**) Western blot bands for PARP1, SIRT1, P62, and LC3 in the hippocampus of each mouse group. (**B–E**) Protein quantitative analysis for each indicator. The data is presented as mean ± standard error, with * P<0.05 and ** P<0.01. n=6.

ELISA results confirmed that esketamine can enhance surgery-induced neuroinflammation. To further examine the relationship between this effect and the activation of neuronal autophagy at the molecular level in esketamine, western blot analysis was conducted on the hippocampal tissue of mice from the previous experiment. The relevant protein expression levels were quantified using Image J software, and the results are depicted in Figure 2.

When compared to the control (C) group, the surgery (S) group exhibited a significant increase in PARP1 expression levels and a significant decrease in SIRT1 expression levels. Conversely, the group receiving esketamine post-surgery (SE group) revealed significantly reduced PARP1 expression and elevated SIRT1 expression levels compared to the S group (P<0.05) (Fig. 2A–C). These findings indicate that exploratory laparotomy activates PARP1 expression while suppressing SIRT1 expression in the hippocampus, whereas esketamine administration can inhibit PARP1 expression and enhance SIRT1 expression. Simultaneously, the expression levels of autophagy-related proteins were examined. The S group revealed a significant increase in the expression of P62, a marker of impaired autophagy, and a significant decrease in LC3 expression levels compared to the C group. In contrast, the SE group exhibited decreased P62 levels and increased LC3 expression when compared to the S group. This indicates that exploratory laparotomy suppresses neuronal autophagy in the hippocampus, while esketamine can reverse this suppression, thereby enhancing neuronal autophagy. In summary, exploratory laparotomy activates PARP1 expression, which inhibits neuronal autophagy, exacerbating neuroinflammation and leading to cognitive dysfunction. Esketamine, by inhibiting PARP1, promotes neuronal autophagy, thereby alleviating neuroinflammation and enhancing cognitive dysfunction.

### Esketamine inhibits lipopolysaccharide-mediated release of pro-inflammatory cytokines and PARP1 expression

**Fig. 3:**
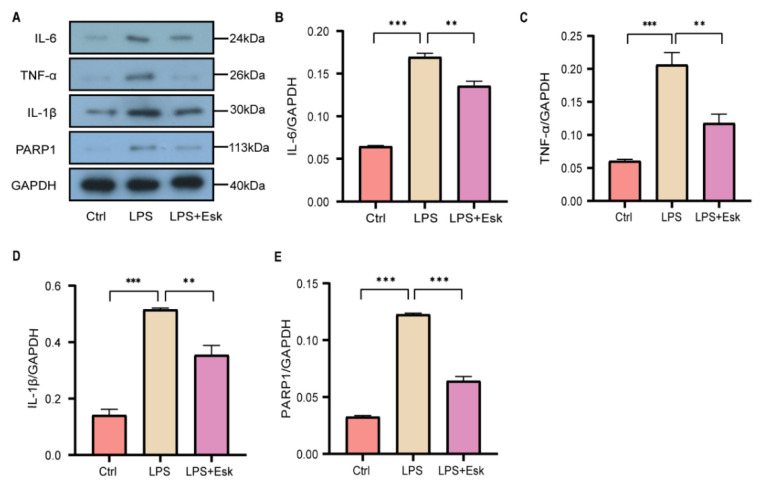
LPS-induced microglial inflammation and the expression of inflammatory factors and PARP1 following esketamine treatment. (**A**) Western blot bands for IL-6, TNF-α, IL-1β, and PARP1. (**B–D**) Protein quantification of each inflammatory factor. (**E**) Protein quantitative analysis of PARP1. These findings suggest that esketamine inhibits PARP1 expression while improving the LPS-mediated microglial inflammatory response. The data is presented as mean ± standard error, with ** P<0.05 and *** P<0.01. Lipopolysaccharides (LPS) and esketamine (Esk).

Given that microglia are the primary effector cells involved in neuroinflammation, the effect of esketamine on microglial inflammation was examined using an LPS-induced inflammation model in BV2 microglial cells [23]. BV2 cells were treated with LPS to induce an inflammatory response, followed by treatment with esketamine. Western blot analysis was used to assess the expression changes of inflammatory markers (IL-6, TNF-α, IL-1β) and PARP1, with the results presented in Figure 3. Figures 3A–D depict the changes in inflammatory factors. The LPS group demonstrated significantly elevated levels of IL-6, TNF-α, and IL-1β compared to the control (Ctrl) group (P<0.01). In contrast, the LPS+Esk group exhibited significantly reduced levels of these inflammatory markers when compared to the LPS group (P<0.05), indicating that esketamine mitigates microglial inflammation. Furthermore, Figures 3A and 3E depict changes in PARP1 expression. The LPS group revealed a significant increase in PARP1 levels compared to the Ctrl group, while the LPS+Esk group revealed a significant reduction in PARP1 expression following esketamine treatment (P<0.01). This indicates that microglial inflammation induces PARP1 activation, which can be inhibited by esketamine. Overall, these findings demonstrate that esketamine effectively reduces microglial inflammation through the downregulation of PARP1 expression.

### Esketamine mediates the PARP1-SIRT1 pathway to regulate autophagy and enhance neuroinflammation

**Fig. 4:**
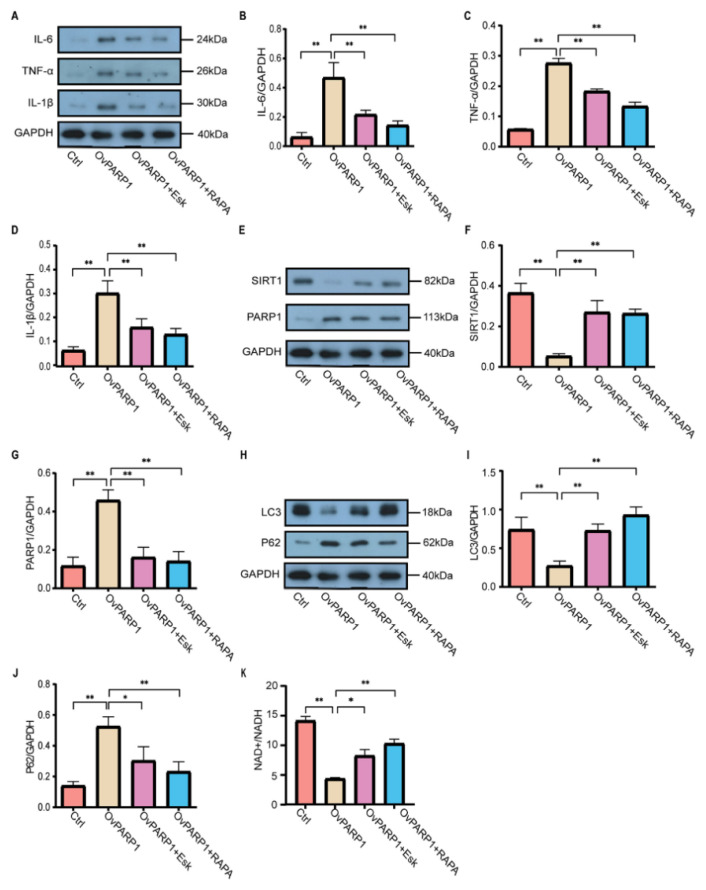
Esketamine inhibits PARP1 overexpression, promotes autophagy via the PARP1-SIRT1 pathway, and improves neuroinflammation. (**A**) Western blot analysis of inflammatory cytokines, revealing bands for IL-6, TNF-α, and IL-1β. (**B–D**) Quantitative analysis of IL-6, TNF-α, and IL-1β protein levels, as determined by Western blot, expressed as mean ± standard error (SE). (**E**) Western blot analysis of SIRT1 and PARP1 protein expression. (**F–G**) Quantitative analysis of SIRT1 and PARP1 protein levels, presented as mean ± SE. (**H**) Western blot bands for autophagy-related proteins LC3 and P62, indicating autophagy activity. (**I–J**) Quantitative analysis of LC3 and P62 protein levels, shown as mean ± SE. (**K**) Biochemical measurement of NAD^+^/NADH content in different treatment groups, reflecting cellular energy status. OvPARP1 refers to BV2 cells transfected with a PARP1 overexpression plasmid. OvPARP1+Esk represents BV2 cells transfected with a PARP1 overexpression plasmid for 24 h, followed by culture in medium containing 5 μg/ml esketamine. OvPARP1+RAPA refers to BV2 cells transfected with a PARP1 overexpression plasmid for 24 h, then cultured in medium containing 100 nmol/l rapamycin. Data are represented as mean ± SE, with * P<0.05 and ** P<0.01 indicating statistically significant differences.

To further examine the mechanism by which esketamine modulates neuroinflammation through the regulation of PARP1 expression, a microglial OvPARP1 model was established. The results, presented in Figure 4A–D, demonstrate that elevated PARP1 expression (OvPARP1 group) is associated with increased levels of the inflammatory factors IL-1β, IL-6, and TNF-α. Following esketamine treatment, the expression of these inflammatory markers decreased. A similar reduction in inflammatory factor levels was observed with the administration of the autophagy agonist, rapamycin. These findings indicate that OvPARP1 induces microglial inflammation, while esketamine effectively suppresses the inflammation triggered by elevated PARP1 levels. Rapamycin also exhibits anti-inflammatory effects in this context. Previous research has indicated that PARP1 can influence processes such as cellular senescence, stress response, and inflammation through its regulation of SIRT1 [17,24].

In our study, SIRT1 expression was assessed, with the results depicted in Figure 4E–G.

The OvPARP1 group exhibited a significant reduction in SIRT1 expression compared to the Ctrl group (P<0.05). Conversely, the OvPARP1+Esk group revealed a significant increase in SIRT1 levels when compared to the OvPARP1 group (P<0.05), indicating that esketamine inhibits PARP1 and consequently activates SIRT1 expression. Activation of SIRT1 promotes the deacetylation of LC3, thereby initiating autophagy [25]. LC3 and P62 are key autophagy-related proteins commonly analyzed in cellular studies [26,27]. According to Figure 4H–J, OvPARP1 led to a decrease in LC3 expression and an increase in P62 levels, indicating that OvPARP1 suppresses autophagy in microglia. Esketamine treatment of OvPARP1 cells reversed this effect, increasing LC3 levels and reducing P62 levels, similar to the effects observed with rapamycin treatment. This indicates that esketamine, similar to rapamycin, can enhance autophagy in microglial cells. Considering that SIRT1 functions as an NAD^+^-dependent protein deacetylase, and that PARP1 activation depletes NAD^+^, NAD^+^ levels were measured in the study [28]. As depicted in Figure 4K, the OvPARP1 group exhibited significantly reduced NAD^+^ levels compared to the control group, indicating that excessive PARP1 activation leads to NAD^+^ depletion. This finding is consistent with the research by Covarrubias *et al.*, further supporting the inhibitory effect of PARP1 on SIRT1 [29]. In conclusion, the data indicate that PARP1 contributes to neuroinflammation by inhibiting SIRT1-mediated autophagy. Esketamine mitigates neuroinflammation by inhibiting PARP1 expression, thereby activating the PARP1-SIRT1 pathway to promote autophagy and enhance neuroinflammation.

### Esketamine inhibits PARP1 expression and activates microglial autophagy

**Fig. 5:**
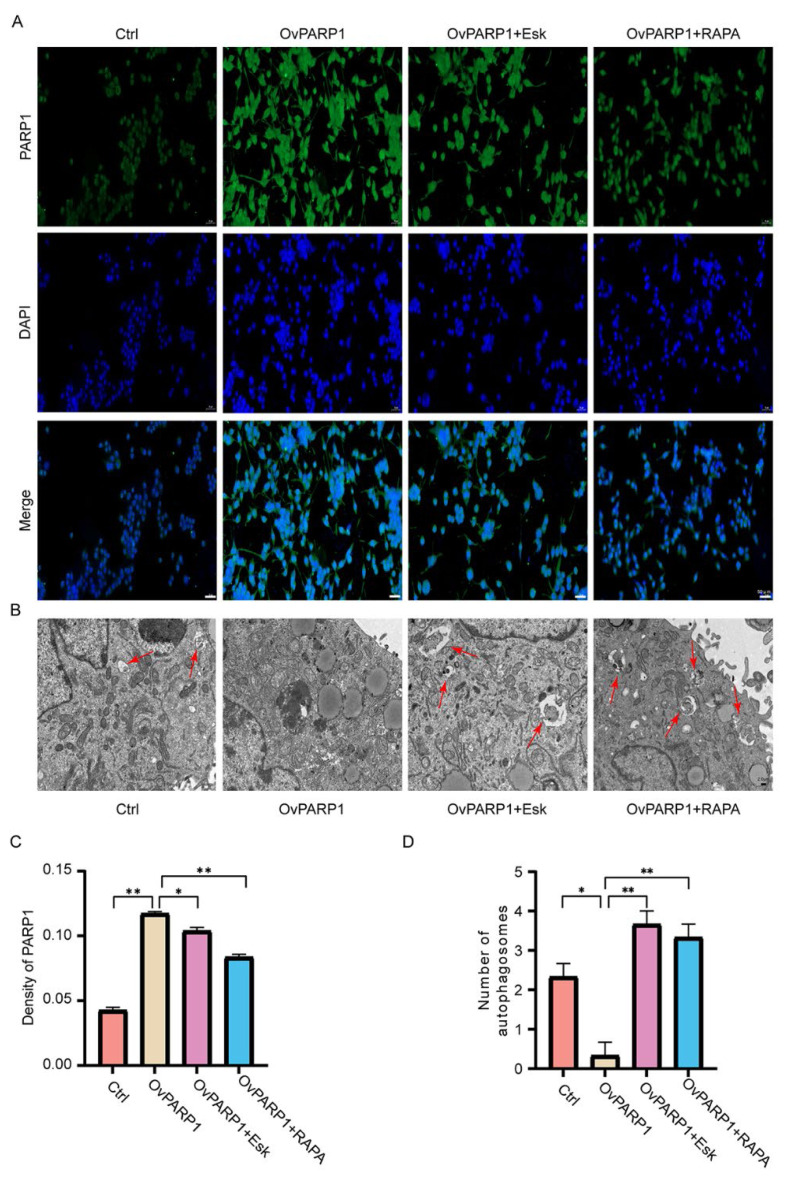
Esketamine inhibits PARP1 and promotes autophagy. (**A**) PARP1 imaging in each group using an immunofluorescence microscope and DAPI staining of the cell nuclei (50 μm). (**B**) Autophagosomes were observed in cells from each group using a transmission electron microscope (2.0 μm). (**C**) Analysis of the average optical density of PARP1. (**D**) A comparison of autophagosomes in cells from each group. * P<0.05, ** P<0.01.

By using immunofluorescence microscopy and transmission electron microscopy, we obtained a more direct visualization of the relationship between PARP1 expression and cellular autophagy. The findings are presented in Figure 5. Figure 5A depicts the expression of PARP1 in microglia observed *via* immunofluorescence microscopy, while Figure 5C depicts the corresponding quantitative analysis of average optical density values using Image-Pro Plus 6.0 software. The analysis reveals that the average optical density of PARP1 in the OvPARP1 group is elevated compared to the control group. In contrast, the OvPARP1+Esk group exhibits a significant reduction in average optical density of PARP1 compared to the OvPARP1 group, indicating that esketamine effectively suppresses PARP1 expression.

To assess the autophagy status of microglia in each group, transmission electron microscopy was used, as depicted in Figure 5B. The number of autophagosomes serves as an indicator of autophagy levels, with red arrows marking typical autophagosomes. Figure 5D quantifies the autophagosome counts for each group. The results indicate that the OvPARP1 group displays a significant decrease in the number of autophagosomes compared to the control group (P<0.05), reflecting suppressed autophagy. Conversely, the OvPARP1+Esk group reveals a significant increase in autophagosome counts compared to the OvPARP1 group (P<0.01), indicating that esketamine can enhance autophagy. A similar increase in autophagosome numbers is observed in the OvPARP1+RAPA group, indicating that esketamine, akin to rapamycin, promotes autophagy. In summary, esketamine inhibits PARP1 expression and activates cellular autophagy.

## Discussion and Conclusion

**Fig. 6:**
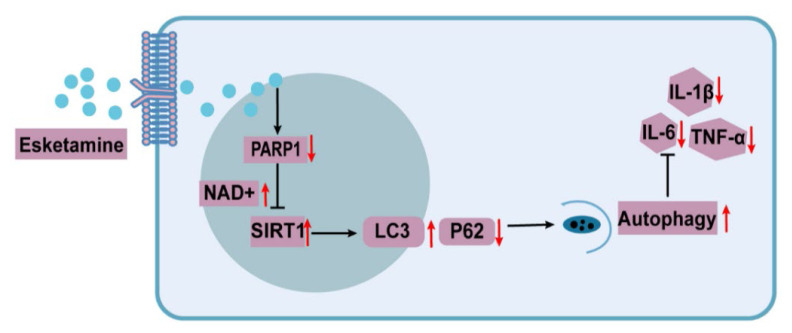
Schematic representation of the mechanism by which esketamine ameliorates neuroinflammation.

POCD is associated with impairments in memory and attention, underscoring the importance of exploring its underlying mechanisms to inform clinical treatment. This study reveals the mechanism through which esketamine improves POCD in a mouse model, as depicted in Figure 6. Specifically, esketamine inhibits PARP1 expression, reduces NAD^+^ depletion, enhances SIRT1 activity, and subsequently activates microglial autophagy, which ameliorates neuroinflammation and improves POCD symptoms in mice.

Neuroinflammation is recognized as a key mechanism contributing to the development of POCD [30]. Surgical trauma can trigger the release of pro-inflammatory cytokines that are capable of crossing the blood-brain barrier, leading to central nervous system inflammation [31]. Microglia, the resident immune cells of the brain, play a significant role in influencing cognitive and behavioral functions [32]. However, the extent to which microglia mediate neuroinflammation in the context of POCD remains to be fully established.

Previous studies have indicated that dexmedetomidine can mitigate microglial inflammation in cases of postoperative neurocognitive disorders, and ulinastatin alleviates POCD by reducing neuroinflammation [33,34]. Research on the role of esketamine in reducing neuroinflammation, however, remains limited. In this study, esketamine inhibited the expression of pro-inflammatory cytokines, including IL-1β, IL-6, and TNF-α, within the hippocampus of mice, leading to enhanced cognitive function post-surgery. This improvement was evidenced by a reduction in escape latency, an increase in the time spent in the target quadrant, and a greater number of platform crossings during the spatial exploration phase of the water maze test. Additionally, *in vitro* experiments demonstrated that esketamine can alleviate LPS-induced microglial inflammation. These findings indicate that the application of esketamine in anesthesia and surgical contexts may effectively suppress central nervous system inflammation, thereby contributing to the mitigation of POCD.

Esketamine, an isomer of ketamine with reduced side effects, was initially introduced for the clinical management of anxiety and depression [35]. However, standard doses of esketamine can still induce adverse effects such as hallucinations and hypertension, leading to increased interest in the potential benefits of low-dose esketamine. Previous studies have demonstrated that ketamine inhibits the expression of LPS-induced inflammatory factors in BV2 microglial cells in a dose-dependent manner, with significant reductions in inflammation observed at 10 μg/ml [36]. Given that esketamine is approximately twice as potent as ketamine, a 5 μg/ml dose of esketamine was used in this study for treating BV2 cells, which effectively reduced inflammation.

Autophagy is a key process involved in various pathophysiological conditions, and microglial autophagy is particularly important for maintaining neuronal survival and function [37,38]. This research discovered a novel mechanism through which esketamine regulates the PARP1-SIRT1 autophagy pathway, providing new insights for the fundamental understanding of cognitive dysfunction.

PARP1 is a key member of the PARP family, known for its rapid activation during inflammatory conditions and its role in promoting the expression of pro-inflammatory mediators [39]. The interaction between PARP1 and SIRT1 is implicated in the regulation of DNA damage repair, mitochondrial function, and inflammatory responses [40]. Through *in vivo* and *in vitro* experiments, this study demonstrated that esketamine modulates PARP1 to activate autophagy, thereby mitigating neuroinflammation. These findings indicate a potential therapeutic target for improving POCD. However, there were also several limitations in this study. The investigation of this study into the mechanisms of autophagy was limited, focusing primarily on short-term postoperative cognitive outcomes in a small sample size. Further research is needed to understand the impact of esketamine on long-term outcomes in perioperative neurocognitive disorders, as well as to assess its effects on other brain regions and neural cell types, including astrocytes and neurons. New object recognition experiments to further explore whether the protective effect of ketamine on long-term cognitive function after surgery is sustained will be conducted in the future, too. This study only verified the PARP1-SIRT1-autophagy pathway in one direction and did not use inhibitors reverse validation of the pathway. Therefore, the study has limitations. Methods of function gain and function loss will be combined to more appropriately verify the mechanistic conclusions. In addition, translational studies will be needed to explore optimal dosing, timing, and potential off-target effects in clinically relevant models.

In summary, this study indicates that esketamine ameliorates early POCD in mice by inhibiting PARP1 expression and modulating the PARP1-SIRT1 signaling pathway, thereby activating autophagy and reducing neuroinflammation. The significance of these findings lies in the identification of the role of esketamine in regulating PARP1 expression, thereby providing a foundation for exploring potential therapeutic targets for POCD. Targeting the PARP1-SIRT1 axis presents a promising strategy for POCD management, providing new clinical perspectives for treatment approaches.

## Figures and Tables

**Table 1:** Inflammatory factor levels in mouse hippocampal brain tissue in each group (pg/mg, n=6, mean ± SD).

Group	IL-1β	IL-6	TNF-α
*C*	4.05±0.57	7.88±1.61	46.32±9.25
*N*	4.10±0.95	8.12±2.12	52.70±10.60
*S*	7.52±0.51**	15.72±0.86**	104.06±9.61**
*SE*	5.43±1.20**,##	9.72±1.43^*^,##	72.89±16.13**,##

** Compared with C group,

## compared with S group.

P<0.05 indicates statistically significant difference.

## Abbreviations

BDNF: Brain-derived neurotrophic factor
BSA: Bovine serum albumin
C/Ctrl: Control group
DAPI: 4′,6-diamidino-2-phenylindole
ECL: Enhanced chemiluminescence
ELISA: Enzyme-linked immunosorbent assay
Esk: Esketamine
FBS: Fetal bovine serum
IL-1β: Interleukin 1 beta
IL-6: Interleukin-6
LC3: Light chain 3
LPS: Lipopolysaccharides
MWM: Morris Water Maze
N: Non-surgery group
NAD^+^: Nicotinamide adenine dinucleotide
OvPARP1: PARP1 overexpression;P62, Protein 62
PARP1: Poly ADP-ribose polymerase-1
PVDF: Polyvinylidene fluoride
RAPA: Rapamycin S, Surgery group
SDS-PAGE: Sodium dodecyl sulfate – polyacrylamide gel electrophoresis
SE: Surgery and esketamine given group
SIRT1: Sirtuin 1
TEM: Transmission electron microscopy
TNF-α: Tumor necrosis factor α
TrkB: Tyrosine kinase receptor B
